# Thai district Leaders’ perceptions of managing the direct observation treatment program in Trang Province, Thailand

**DOI:** 10.1186/s12889-016-3341-1

**Published:** 2016-07-28

**Authors:** Jiraporn Choowong, Per Tillgren, Maja Söderbäck

**Affiliations:** 1 School of Health, Care and Social Welfare, Mälardalen University, PO Box 883, SE 721-23 Mälardalen, Sweden; 2 Boromarajonani College of Nursing Trang, Praboromarajchanok Institute for Health Workforce Development (PIHWD), Ministry of Public Health, 92000 Trang, Thailand

**Keywords:** DOT program, Leadership, Phenomenography, PARIHS model, Thailand

## Abstract

**Background:**

Thailand is 18th out of the 22 countries with the highest tuberculosis (TB) burden. It will be a challenge for Thailand to achieve the UN Millennium Development target for TB, as well as the new WHO targets for eliminating TB by 2035. More knowledge and a new approach are needed to tackle the complex challenges of managing the DOT program in Thailand. Contextual factors strongly influence the local implementation of evidence in practice. Using the PARIHS model, the aim has been to explore district leaders’ perceptions of the management of the DOT program in Trang province, Thailand.

**Methods:**

A phenomenographic approach was used to explore the perceptions among district DOT program leaders in Trang province. We conducted semi-structured interviews with district leaders responsible for managing the DOT program in five districts. The analysis of the data transcriptions was done by grouping similarities and differences of perceptions, which were constructed in a hierarchical outcome space that shows a set of descriptive categories.

**Results:**

The first descriptive category revealed a common perception of the leaders’ duty and wish to comply with the NTP guidelines when managing and implementing the DOT program in their districts. More varied perceptions among the leaders concerned how to achieve successful treatment. Other perceptions concerned practical dilemmas, which included fear of infection, mutual distrust, and inadequate knowledge about TB. Further, the leaders perceived a need for improved management practices in implementing the TB guidelines.

**Conclusion:**

Using the PARIHS framework to gain a retrospective perspective on the district-level policy implementation of the DOT program and studying the leadership’s perceptions about applying the guidelines to practice, has brought new knowledge about management practices. Additional support and resources from the regional level are needed to manage the challenges.

## Background

Tuberculosis (TB) is an emerging global health problem, as can be seen by its unequal distribution in the world. TB is strongly associated with poverty, with 95 % of all tuberculosis cases occurring in developing countries [1]. The United Nations (UN) states in its 2014 report following up on the Millennium Development Goals (MDGs), that although 56 million TB patients were successfully treated between 1995 and 2012, and 22 million human lives were saved, the target for TB (Target 6c) target for TB (Target 6c) would not be reached by 2015. Even more effort is needed to achieve the MGDs for TB [2]. Also, in 2014 the World Health Organization (WHO) highlighted the worldwide TB situation. A new global strategy and targets for the prevention, treatment and control of TB after 2015 were adopted, with a bold vision of a world without tuberculosis. The goal of ending the global tuberculosis epidemic by 2035 will be accomplished through a 95 reduction of TB deaths and a 90 % reduction in TB incidence (or less than 10 TB cases per 100 000 persons) [3].

Thailand is 18th out of the 22 countries with the highest tuberculosis burden in the world. With a population of approximately 67 million, it had in 2012 an estimated prevalence rate of 159 and an incidence rate of 119 cases per 100,000 persons for all forms of TB [4, 5]. The Directly Observed Treatment Short-Course (DOTS) strategy was adopted in Thailand as the core policy of the National Tuberculosis Program (NTP) Guidelines for TB control in 1996 [6]. This strategy consists of five key elements: political commitment, case detection through sputum microscopy, a standardized treatment regimen with directly observed treatment (DOT) for at least the first 2 months, regular drug supply, and a standardized recording and reporting system where the treatment outcome for each patient in evaluated by cohort analysis [7]. The efforts under the DOT program have resulted in an average TB cure success rate of 84, ranging between 74 and 94 % in the country’s 77 provinces [8]. It will be a challenge for Thailand to achieve the UN Millennium Development target for TB, as well as the new targets from the WHO to eliminate TB by the year 2035 [4]. The goal of Thailand’s National Strategic Plan for Tuberculosis Control 2015–2019 is to reduce TB case incidence from 80,000 to 60,000 by 2019 (25 % reduction) [9]. More knowledge and a new approach are needed to deal with the complex challenges of managing the DOT program in Thailand.

### Theoretical framework

It is increasingly recognized that research on implementation in health services should be theory driven. In this study we used the Promoting Action on Research Implementation in Health Services (PARIHS) model as our theoretical framework [9, 10]. The PARIHS model highlights three basic elements of successfully implementing a research-based policy in practice: the nature of evidence, the context in terms of coping with change, and the types of facilitation [10, 11]. The nature of the evidence for the DOT program is based on WHO’s recommendations [7, 12].

Health care practice does not, however, always reflect what is known to be the best practice as identified by research. Contextual factors strongly influence the local implementation of evidence in practice. In this study, which is part of a larger project, the PARIHS model is used to gain a retrospective perspective on the policy implementation of the DOT program at district level. According to the PARIHS model the sub-elements in the context that are useful for research consist of the receptive culture as well as the leadership who will transform the knowledge into practice. In Thailand the facilitators are Village Health Volunteers (VHVs) and family members (FMs) who act as DOT observers in the community. Their experiences will be investigated in another study.

In the PARIHS model, the culture is defined as the “learning organization”. In Thai culture, the health care system has a centralized public health infrastructure. The DOT program has a hierarchical administrative structure in accordance with the NTP guidelines [6]. We focus in this study on the element of leadership: those who are responsible for implementing the DOT program in practice [10, 11].

The NTP implementation in Thailand is decentralized to the districts, which serve as the basic administrative units for TB control. National TB coordinators monitor the NTP implementation in Thailand’s four geographic regions (north, northeast, central, and south). Regional TB centers are integrated into the Regional Office of Disease Prevention and Control (ODPC), and are responsible for monitoring, training, and supervising TB health workers at the provincial and district levels. The Provincial TB Coordinators (PTCs) are responsible for monitoring the NTP implementation in each of the provinces. It is the district leaders [District TB coordinators (DTCs) and TB clinic staff] who are directly responsible for managing and running the TB control program [6]. It has been found in earlier research that some causes of unsuccessful TB treatment arose because the DOT program was led and managed by the healthcare providers [13–16]. There is a lack of comprehensive knowledge regarding the district leaders of the DOT program in Thailand.

Trang province, which was chosen for this study, had a population of about 640,000 in 2013. The estimated prevalence rate was 67 cases and the incidence rate 42 cases per 100,000 persons for all forms of TB. Although the DOT strategy and the NTP guidelines were implemented in all districts, the province has had an average TB cure success rate of 90, ranging between 74 and 100 % in the nine different districts [8]. As highlighted in the PARIHS model, the successes, failures and impacts of the DOT program can be explained in terms of the quality of the contextual elements and leadership. Improving the DOT program management requires understanding how the leaders perceive it. Thus the aim was to explore the district leaders’ perceptions of the management of the DOT program in Trang province, Thailand.

## Methods

### Study design, participants

A qualitative phenomenographic approach with semi-structured in-depth interviews was used to explore different perceptions of the DOT program management [17, 18]. The phenomenographic analysis will generate an outcome space that describes varying perceptions among individual district TB leaders [19].

Purposive sampling was used for the study [17]. District leaders who faced the most new TB cases in their districts were selected because they were considered to have the most experience. Selection criteria included choosing both large and small districts and recruiting men and women from different areas and with different lengths of service to gather a variety of individual experiences [19]. In each of the five districts, one person from the district hospital TB clinic and one person from the district public health office in charge of TB control was selected. The sample size was decided according to the concept of saturation because there is no formal way of determining sample size for qualitative research [20]. The sample comprised five female and five male leaders. Their mean age was 45.4 years (range 37 to 56 years) and they all had at least 1.5 years of experience in TB projects (range 1.5 to 16 years).

### Data collection

The interviews were conducted by the first author at district hospitals and the district public health offices. The interviews were performed in the Thai Language. The first question asked was open ended: “How do you perceive the management of the DOT program for TB patients?” Then, guided by semi-structured questions, the interviews continued to explore their various perceptions. Additionally, probing techniques were employed, using questions like: “Could you describe more about that?”, “Could you give me an example?”, and “What does that mean to you?” These questions were intended to encourage them to clarify and elaborate on their perceptions. To enhance self-awareness, the first author made reflective notes immediately after the interviews in order to document the events, actions/interactions, and thinking processes [20]. Each interview was digitally recorded and lasted approximately 60–90 min.

### Data analysis

The interviews were transcribed verbatim in Thai by the first author. The recordings were listened to several times to ensure complete understanding of the transcripts. The texts were then translated into English, the common language among all the authors. For syntactical accuracy, the transliteration was checked by an expert English writer [21], as well as by the third author to confirm that the translation closely reflected Thai culture. The transcribed text was analyzed using phenomenographic analysis to identify the perceptions, in the form of intended conceptual meanings, expressed in the leaders’ utterances [17–19].

The texts were initially read and re-read in their entirety to create close familiarity with the content. After the initial readings, the researchers looked for similarities and differences in utterances. This utterances were decontextualized, and a tentative grouping was made of similarities and differences in the leaders’ perceptions of managing the TB program. During the analysis process, the authors met several times to discuss and revise the identified perceptions, and to confirm that they were validly derived from the utterances. This process involved several revisitings and re-readings of the transcripts, testing the perceptions against both the immediate context of surrounding statements and the transcript as a whole. Then the final perceptions were constructed within descriptive categories. The set of descriptive categories formed a hierarchical outcome space resulting from the leaders’ perceptions of managing the DOT program (Table 1).

**Table 1:** The outcome space for the district leaders’ perceptions of managing the DOT program in Trang Province, Thailand

(4) Need for improvement	
Motivation	
For district leaders: Extrinsic motivation	
For VHVs: Extrinsic motivation and intrinsic motivation	
In the health system: Community participation	
New practices in the TB guidelines	
District leaders: Having a family member as a mentor	
Health system: Having effective informational materials for village health volunteers	
(3) Practical dilemmas	(2) Achieving a full recovery
Fear of infection	Need for trust
District leaders: Fear of infection	District leaders:
VHVs: Fear of infection	Trust the TB patients and their families
TB patients and their families: Fear of participation restriction	TB patients and their families: Having self-awareness or self-responsibility
Mutual distrust	Needs of care
District leaders:	District leaders: Home visits and follow ups
Don’t trust TB patients and their families	VHVs: Empowering TB patients and their families
VHVs: Unsuitable	TB patients and their families:
TB patients and their families: Don’t trust village health volunteers	Empowerment, caring, and health education
Inadequate knowledge about TB	Necessity of community participation
District leaders: Insufficient knowledge to conduct the DOT program	Good cooperation among stakeholders (district leaders, VHVs, TB patients and their families, villagers)
VHVs: Insufficient knowledge to perform the DOT program	
(1) Compliance with the TB treatment program guidelines	
Management of the DOT program follows the guidelines of the national TB program	
Health education training for village health volunteers	

## Results

The outcome space of the district leaders’ perceptions of managing the DOT program for TB treatment is constructed around four descriptive categories. The first category revealed a common perception among the leaders was that they felt it was their duty to comply, and wanted to comply, with the NTP guidelines when managing and implementing the DOT program in their districts. Further perceptions identified among the leaders concerned how to achieve successful treatment. Another descriptive category involved perceptions pertaining to several practical dilemmas which included fear of TB infection, mutual distrust, and inadequate knowledge about TB. The leaders’ perceptions also differed when it comes to the need to improve both extrinsic and intrinsic motivation, as well as the need for a new method of following the TB guidelines (Table 1). The results, including the sub-categories, are reported in more detail below.

### Compliance with the TB guidelines

Overall the district program leaders perceived compliance with the NTP guidelines that call for monitoring the treatment of TB patients and preventing infection of their families as very important. The district leaders described trying to manage their responsibilities as well as possible. However they could not follow the TB guidelines in every instance, for example when providing treatment for a TB patient who has multidrug-resistant tuberculosis (MDR-TB). One participant said:

*Actually, as soon as the patient has been diagnosed with MDR-TB, the local health care center should be assigned to keep track of any local TB patient who has lived in such and such area. In practice, they would be referred to the provincial hospital. Some patients could not afford the cost of transportation to the hospital. I have personally asked the district public health officer to care for the patients in these cases. I was able to manage it. [TB clinic staff]*

The district TB leaders also described the importance of education in supporting and coaching the VHVs to comply with the guidelines by specifically training them in TB health education. They trained the VHVs not only in groups, but also individually, and described three levels of training. The first level was offered to the provincial TB-control staff by the Regional Office of Communicable Disease Control trainers. The regional staff would then act as teachers and train the district level staff. Consequently, they saw the district level staff as responsible for training the VHVs. These teachers would then arrange full-day intensive courses to train groups of VHVs selected from each village. Finally, they saw the VHVs as observers who controlled the treatment of the TB patients. In their view, to become observers, VHVs agreed to take individual responsibility for monitoring the TB patients and recording their compliance after receiving training. It was told:

*For teaching, I had pictures to show them about how to follow the TB educational program manual. For individuals, I would ask the VHV to come see me or the Tambon health promotion hospital staff. We would then talk about the program; how to care for the TB patient based on the DOT program; how to make the program work by keeping the medication with the VHV and either having the patient walk to the VHV to take the medication or having the VHV bring the medication to the patient; and, after the patient took the medication, completing the record. [TB clinic staff]*

### Achieving a successful cure

The district program leaders also perceived achieving a successful cure as very important. This included ensuring that the TB patients completed their treatment course and did not spread the disease to anyone else in their household or community. Moreover, the district leaders described the necessity of trusting and caring for the TB patients and of community participation, to be able to successfully cure the patient. The district leaders described having to rely on the TB patients to take responsibility for caring for themselves and completing the treatment course. One participant stated:

*If TB patients come here to take it with me every day, they will complete their full course of medications on their own. When they are somewhere else, I have to trust them. [TB clinic staff]*

The district TB leaders described different ways to care for the TB patients that depended on the individual’s personality and how the patient feels and reacts to the disease. Therefore, how to manage the TB patients had to be considered on a “case by case” basis. To care for patients individually, the DTCs also had to cooperate with the PTCs by making follow-up home visits twice a month. Both the coordinators and the VHVs charged with being the patients’ observers have to communicate individually with the TB patients. This includes making sure that the VHVs observe that the TB patient has completely followed the DOT program. One PHO stated:

*When making visits, we actually found that the TB patients and the VHVs were on very close terms. Most of the time, they would just ask “Have you taken your pills yet?” The VHVs won’t watch the patient taking the pill in front of them. In such cases, we have to ask them to strictly follow the DOT guidelines by taking the pills while being watched every time. [DTC]*

Furthermore, the district leaders stressed the importance of community participation and cooperation among networks in the communities, including the Director of the District Hospital, the TB clinic staff, DTC, THPH staff, the VHVs, and the community members or villagers, as stated in the national TB program.

*It was believed that as long as the community understands the seriousness of TB in relation to themselves, we would have a helping hand. It also helped ease our work. [DTC]*

### Practical dilemmas

Although the district leaders generally perceived a need to comply with the NTP guidelines to reach the goals of DOT treatment, they also described practical dilemmas which included a general fear of infection, mutual distrust, and inadequate knowledge about TB.

The district leaders described a general fear of being infected by TB both among themselves and VHVs, as well as among family members and villagers. Most of the TB clinic staff did not like dealing with TB patients because it is a communicable disease. So when the District leaders were assigned to a TB case, they had to work out how to cope with it and to protect themselves. They perceived the VHVs as also afraid of getting infected, despite having gone through a TB education program and learning how to protect themselves. Moreover, they explained that the TB patients were afraid, and did not want others in their personal surroundings and village to know about their diagnosis for fear of being ostracized and stigmatized. Two district leaders shared the following:

*Oh, it was so bad when I heard “tuberculosis”. What immediately came to my mind was that it was contagious. [TB clinic staff]*

Another of the interviewees said:

*However, the TB patients were very likely to refuse to transfer out to another health center… because they didn’t want anyone to know they were infected. They kept it secret. [TB clinic staff]*

Continuing, the district leaders described how the “mutual distrust” affected the TB patients’ willingness to participate in, and comply with, the treatment program. They told how TB patients mistrusted VHVs, because patients had caught VHVs violating their confidentiality by discussing their situation with other villagers. Then the TB patients would want to take their medication without an observer. Also, the district leaders declared they did not trust TB patients. They explained that Thais were afraid to argue with physicians and would not go to the hospital unless they were seriously ill. The patients did not like having someone watching while took their medication. The district leaders stated that this was the reason why they did not believe that relatives could be trusted to monitor whether the TB patients were taking their medications. A participant offered the following opinion:

*Thai people love to be easy-going about everything and don’t take care of themselves well enough. When they feel better, they think they are cured and decide on their own to stop taking their medication. [TB clinic staff]*

Another practical dilemma was inadequate knowledge about TB. Such a lack of knowledge was perceived when new district leaders were in charge of TB cases at the district level. This made it difficult for them to maintain continuity in understanding the clinical signs of TB and entering and reporting data. Although the VHVs had been trained to be individual mentors, and manuals or handouts were available to them, their knowledge about TB was still sometimes inadequate. So, when they returned to the households in the villages, they were less likely to successfully fulfill their role as mentors. The district leaders explained that the VHVs served an important function as mentors; they must be willing to sacrifice and be determined to do what must be done. One participant said:

*I was not very knowledgeable about the TB clinic because I was new to the job. I had never been trained in-depth about clinical TB information. [DTC]*

Another interviewee stated:

*After completing their training, the VHVs were unable to apply what they had learned in the program to caring for the TB patients and their families. [TB clinic staff]*

### The need for improvement

Some district leaders described a need to make changes to improve the management system. These changes included improving both the extrinsic and intrinsic motivation among the actors involved in the DOT management system, and finding a new method of practicing the TB guidelines. District leaders’ extrinsic motivation was centered on DOT program funding. They wanted to receive more financial support to cover their expenses, as an incentive. They explained that only three of the ten districts received financial support from the Global Fund. In the other seven, the district leaders had to pay out of their own pockets for either pre-paid phone cards for follow-up calls and monitoring of TB patients or for patients’ transportation expenses and meals.

Furthermore, another important motivational improvement that the district leaders emphasized was organizing a system to honor and boost the morale of VHVs who take good care of TB patients. One said:

*I believe we should provide VHVs with knowledge and empower them from time to time. That must be okay. For example, we should assist them financially. [TB clinic staff]*

Another said:

*I think we should recognize the VHVs, such as by awarding the “aoh soh moh” (VHV in Thai) a certificate of appreciation. [DTC])*

The district leaders described a need for improved motivation to collaborate with the community, because they have to work with members of other networks active in the communities. The leader said:

*I would find a way to reach a “Memorandum of Understanding” with the village fund in order to have them take the TB activities under their management. [DTC]*

However, when district leaders described how the management system could be improved, they also mentioned making changes to allow for more individuality in the DOT program. They expressed that sometimes district leaders need to accept that family members act as observers, even though the regional policy states that a family member or relative should only be an observer as a last resort. The district leaders said that they have to consider the needs of individual TB patients because patients differ greatly in terms of who they are, how ill they feel, and how they react to their illness. In cases where the family is close-knit and warm, family members would be able to care for each other and could be good observers. They said:

*Well, if the manual could have possible solutions for allowing a family member to serve as a mentor with the approval of the regional TB center, that would be excellent because right now the center ranks it as the third option. [TB clinic staff]*

The district leaders said that although the VHVs were trained to follow the TB educational program manual (provided by Teacher A-C), some were unable to apply what they had learned about observing and caring for the TB patients. The district leaders also saw a need for better guidance to improve the VHVs’ understanding of their duties and to enable TB patients to practice self-responsibility. They also believed that if the TB educational program manual were more focused on self-care and patient contact, it would empower TB patient to practice self-responsibility. They stated:

*I would like to see a manual that works not only for me, but also for the VHVs, TB patients, and family members. The information should be laid out in easy to understand, practical, lay terms. For example, what a TB patient should do to improve or recover completely. [DTC]*

## Discussion

This study has used the PARIHS framework to get a retrospective perspective on the policy implementation of the DOT program on district level in Trang province, Thailand. An important sub-element in the context is the leadership, who must transform the knowledge and guidelines into actual practice. The district program leaders’ perceptions about the management of the DOT program exhibited a great deal of variety, and formed a hierarchical outcome space. The study found that the district TB leaders managed the DOT program and implemented it by complying with the guidelines, which led to successful cures but also to practical dilemmas. Other perceptions among the district leaders demonstrated a need to make changes to improve the DOT program management in their districts.

The structural health care system in Thailand has a centralized public health infrastructure. The DOT program has a hierarchical administrative structure in accordance with the NTP guidelines. Compliance with hierarchical structure is of importance in Thai culture [6] and the district leaders expressed the importance of managing DOT program in accordance with the NTP guidelines.

However, in managing the DOT program, the district leaders saw a number of practical dilemmas. These included fear of being infected themselves and their discovery that the village health volunteers were also afraid. There was also fear among the TB patients who did not want others to know about their illness, because of the risk of being ostracized. Another dilemma was that when district leaders, VHVs, villagers, patients and family members all lack adequate TB knowledge and skills, they do not feel confident about conducting awareness-raising efforts in their communities. Other studies have found that when communities lack a clear understanding of how TB spreads and is treated, stigmatization of persons with TB is more common. Prior studies on TB stigma describe fear of stigmatization as a significant barrier to early DOT treatment and compliance [6, 15].

Reducing the fear of infection and the mistrust that serves as a barrier to conducting TB prevention and treatment efficiently, and providing information emphasizing that TB is curable, need to be done without scaring people. Earlier research has shown that enhanced support for education and stigma reduction activities will improve the practicality of the TB guidelines for VHVs/TB patients and their families in the province and promote their contact with health services [12, 15, 22].

Thus, a challenge that the district leaders expressed was that they wanted the DOT observers to trust the TB patients, whom they perceived as wanting to take personal responsibility for completing their treatment course. Previous studies show that it is possible to build trust in the relationships between TB patients and DOT observers [23, 24]. This supports previous findings that having a good relationship with patients is indicative of a good therapeutic alliance, that is, a carer-patient relationship characterized by trust, empathy, and positive regard [22]. Further, other research shows that another way to reduce mistrust would be if the specific DOT observer could be chosen by the TB patient [14, 23, 25, 26]. Thus, in this study the district leaders expressed a preference for having family members as mentors.

Moreover, the district leaders expressed that they wanted the DOT observers to care for the TB patients on a “case by case” basis, because this may help in providing more patient-centered care. By cultivating a close relationship with the patient, an observer can gain crucial information for understanding the individual TB patient’s needs [24].

Another practical dilemma that the district leaders perceived when managing the program was that new district leaders as well as the DOT observers lack practical skills and knowledge about TB. To make DOT observers better capable of supporting TB patients, the leaders wanted them to have sufficient training to give medications, understand side-effects, and explain to patients when they have symptoms and why they have to go to for a check-up. As also found in other research, leaders took it for granted that if the DOT observers understand their roles they will perform well in tuberculosis prevention and control, and be able to carry out their responsibilities in a suitable and effective manner [12]. Thus, the leaders at the district level perceived themselves as responsible for training the VHVs.

In Thailand the VHVs serve as the backbone of community-based public health services, and are intended to act as a two-way link between communities and the health care system. The VHVs are expected to provide a wide range of services to families covering a wide range of health care and social support – for instance breast feeding and home based care – in addition to being DOT observers and providing TB education to the patients and their family members [23, 27].

When it comes to improving the DOT program management, most district leaders are aware of both extrinsic and intrinsic motivational factors. According to a prior study, human motivation is a fundamental component of a learner’s willingness to seek and gain knowledge [23]. At present, the Thai NTP’s budgetary resources are provided by the Global Fund, and only cover some districts [8]. There is no system in place to ensure budgetary improvement. In order to stimulate certain behaviors and intrinsic responses by addressing needs, wants, and motivations, the districts have requested support for all of the districts. However, some research shows that we can stimulate not only lay workers’ intrinsic motivation but also their extrinsic motivation, through such things as payment for work done, to ensure the sustainability of TB care provided by the community member/healthcare worker [28]. Furthermore, the leaders perceived a need for a stronger focus on community participation, motivation, and social support, which can be defined as an empowerment approach. It is known that a community empowerment approach can help individuals become knowledgeable about and responsible for their own health. A similar result was also found in a Thai study on improving the DOT program [29].

Some district leaders need to improve program implementation by having effective materials for VHVs. The NTP guidelines were developed and applied in the southern region’s districts by the Bureau of Tuberculosis, Department of Disease Control, Ministry of Public Health. They were meant for everyone acting as DOT observers (health care workers, public health officers, or other responsible persons) [12]. At present, there is no exact way of translating NTP policy guidelines to match the actual conditions at the district level in each province. The different contexts and practice guidelines require modification before they can develop into established practices. Therefore, the district leaders proposed new TB practice guidelines for the VHVs.

### Strengths and limitations

The trustworthiness of the findings was strengthened by triangulation involving multiple researchers in the sensitive translation and analysis processes. It has been shown that a translator who fully understands study participants’ culture and language will reduce potential threats to the validity of the information given [21, 30]. The authors adjusted and re-checked the text translation, from and to the native language, step by step, until consensus was reached. [31]. In addition, this study was reported according to the consolidated criteria for reporting qualitative research (COREQ) guideline. Thus, we have ensured the quality of reporting in this article [32]. Some limitations of our study include the small number of participants, which means that the findings cannot be generalized. We have ensured a valid representation of the perceptions of the district leaders about the subject matter, which will make the findings transferable to other provinces in Thailand and relevant for understanding the experiences and perceptions of leaders there.

## Conclusion

It will be a challenge for Thailand to achieve the UN Millennium Development target for TB. Using the PARIHS framework to gain a retrospective perspective on the district-level policy implementation of the DOT program, and studying the leadership’s perceptions about transforming the guidelines into practice, has brought new knowledge about management practices. The district leaders faced practical dilemmas and challenges in effectively managing the DOT program and TB control efforts.

Improving both extrinsic and intrinsic motivation, and incorporating new practices into the TB guidelines, are essential steps in solving the practical dilemmas. Additional support and resources from the regional level, which is responsible for the provincial and district levels, will be required to manage the challenges. Further study is needed to obtain knowledge from the TB patients themselves as well as from the DOT observers, facilitators, VHVs and family members, who are expected to help and encourage TB patients to go through the DOT program and be cured.

## Abbreviations

DOT, directly observed treatment; DOTS, Directly observed treatment short-course; DTCs, District TB Coordinators; FM, family member; MDGs, Millennium Development Goals; MDR-TB, multidrug-resistant tuberculosis; NTP, National Tuberculosis Program; ODPC, Regional Office of Disease Prevention and Control; PARIHS, Promoting Action on Research Implementation in Health Services; PTCs, Provincial TB Coordinators; TB, tuberculosis; UN, United Nations; VHV, village health volunteer; WHO, World Health Organization
